# Key dimer interface residues impact the catalytic activity of 3CLpro, the main protease of SARS-CoV-2

**DOI:** 10.1016/j.jbc.2022.102023

**Published:** 2022-05-11

**Authors:** Juliana C. Ferreira, Samar Fadl, Wael M. Rabeh

**Affiliations:** Division of Science, New York University Abu Dhabi, Abu Dhabi, United Arab Emirates

**Keywords:** COVID-19, SARS-CoV-2, 3-chymotrypsin-like protease, 3CLpro, thermodynamic stability, kinetic characterization, aSEC, analytical size-exclusion chromatography, 3CLpro, 3C-like protease, COVID-19, coronavirus disease 2019, DMSO, dimethyl sulfoxide, DSC, differential scanning calorimetry, MD, molecular dynamics, β-ME, β-mercaptoethanol, nsp, nonstructural protein, SARS-CoV-2, severe acute respiratory syndrome coronavirus 2, TCEP, Tris(2-carboxyethyl)phosphine

## Abstract

3C-like protease (3CLpro) processes and liberates functional viral proteins essential for the maturation and infectivity of severe acute respiratory syndrome coronavirus 2, the virus responsible for COVID-19. It has been suggested that 3CLpro is catalytically active as a dimer, making the dimerization interface a target for antiviral development. Guided by structural analysis, here we introduced single amino acid substitutions at nine residues at three key sites of the dimer interface to assess their impact on dimerization and activity. We show that at site 1, alanine substitution of S1 or E166 increased by twofold or reduced relative activity, respectively. At site 2, alanine substitution of S10 or E14 eliminated activity, whereas K12A exhibited ∼60% relative activity. At site 3, alanine substitution of R4, E290, or Q299 eliminated activity, whereas S139A exhibited 46% relative activity. We further found that the oligomerization states of the dimer interface mutants varied; the inactive mutants R4A, R4Q, S10A/C, E14A/D/Q/S, E290A, and Q299A/E were present as dimers, demonstrating that dimerization is not an indication of catalytically active 3CLpro. In addition, present mostly as monomers, K12A displayed residual activity, which could be attributed to the conspicuous amount of dimer present. Finally, differential scanning calorimetry did not reveal a direct relationship between the thermodynamic stability of mutants with oligomerization or catalytic activity. These results provide insights on two allosteric sites, R4/E290 and S10/E14, that may promote the design of antiviral compounds that target the dimer interface rather than the active site of severe acute respiratory syndrome coronavirus 2 3CLpro.

Coronaviruses cause acute infections of the upper and lower respiratory tracts with mild to lethal symptoms. One of the most prominent coronaviruses is severe acute respiratory syndrome coronavirus 2 (SARS-CoV-2), the virus responsible for coronavirus disease 2019 (COVID-19), which has caused millions of infections and deaths worldwide as well as major social and economic unrest (1, 2). SARS-CoV-2 is one of the largest positive-sense single-stranded RNA viruses and belongs to the genus *Betacoronavirus*, which also includes SARS-CoV and Middle East respiratory syndrome coronavirus (3, 4, 5). The main protease of SARS-CoV-2, 3C-like protease (3CLpro), is one of two proteases responsible for processing two polypeptides, polyprotein 1a and polyprotein 1ab, to liberate 16 nonstructural proteins (nsps) (6, 7). The nsps are important for replication, transcription, and virus recombination during infection. Inhibiting the catalytic function of the proteases blocks the release of the nsps and the progression of COVID-19, making SARS-CoV-2 3CLpro an attractive target for the design of broad-spectrum antivirals against COVID-19 (8, 9).

The 3CLpro proteases of various coronaviruses have identical structural folds. 3CLpro forms a homodimer in which the two monomers are arranged perpendicular to each other. Studies of 3CLpro from different coronaviruses have suggested that dimerization and interactions at the dimer interface are essential for catalytic activity (10, 11, 12, 13, 14, 15, 16). Each monomer is split into three domains. Domains I (residues 10–96) and II (residues 102–180) form a chymotrypsin-like folding scaffold containing six- and five-stranded antiparallel β-barrel structure, respectively (Fig. 1*A*) (17). The globular helical C-terminal domain III (residues 200–303) comprises a cluster of five α-helices connected to domain II by a long loop (residues 181–199). In 3CLpro of SARS-CoV, domain III is important for dimerization and formation of the active 3CLpro protease (18). The seven N-terminal residues form the N-finger, a long loop that extends from domain I of one monomer toward domain III of the other monomer. The N-finger mediates multiple dimer interface interactions between domains I and II of both monomers. Some of these contact points of the N-finger are important for the enzymatic activity of 3CLpro of SARS-CoV, and Arg4 is critical for its dimerization (19, 20, 21, 22, 23).

**Figure 1:**
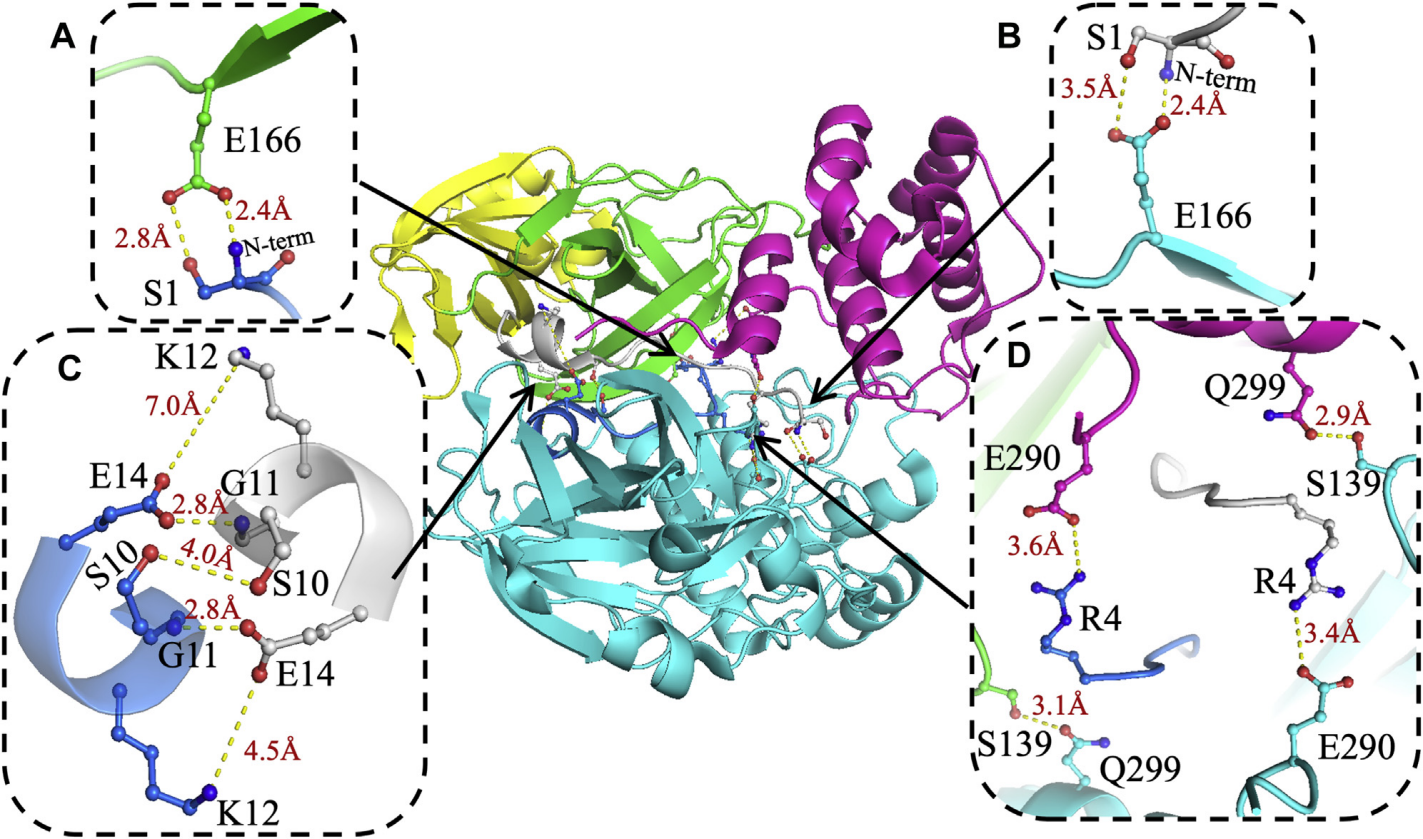
**Dimer interface interactions of SARS-CoV2 3CLpro.** One monomer is shown in *cyan*, and the other monomer is colored by domain: N-finger in *blue*, domain I in *yellow*, domain II in *green*, and domain III in *pink*. Three sites of interactions were identified as important for 3CLpro dimer interface formation. *A* and *B*, at the first site, Glu166 in domain II interacts with the N terminus and side chain of Ser1 in the other monomer to form two points of contact between the two monomers. *C*, at the second site, the one-turn α-helix (residues 11–14) at the end of the N-finger of one monomer interacts with the same region of the other monomer to form a single contact point in the dimer interface. The side chains of the two Ser10 residues form intermolecular H-bonding interactions. Long-distance ionic interactions may occur between Lys12 of one monomer and Glu14 of the other monomer. *D*, the third site includes four residues. Arg4 of the N-finger interacts with Glu290 in domain III of the other monomer. In addition, Ser139 in domain II of one monomer interacts with Gln299 in domain III of the other monomer. The figure was generated using Protein Data Bank code 6WTM and PyMol (Schrodinger LLC) (31). 3CLpro, 3C-like protease; SARS-CoV-2, severe acute respiratory syndrome coronavirus 2.

The active site of 3CLpro is located in a cleft between domains I and II. The cleft contains six subsites (S1–S6) that correspond to P1–P6 of the peptide substrate. The most important and conserved subsite of 3CLpro is S1, which binds P1 (Gln) of the peptide substrate. In S1, the oxyanion hole formed by the main chain amides of the Ser139–Leu141 loop stabilizes the thiohemiketal tetrahedral intermediate formed upon the nucleophilic attack of the thiolate ion of Cys145 on the peptide carbonyl carbon of the substrate (11, 17, 18, 24, 25, 26, 27, 28, 29). His41 and Cys145, which are part of domains I and II, respectively, form a catalytic dyad in which His41 deprotonates the thiol side chain of Cys145 to facilitate its nucleophilic attack on the backbone carbonyl carbon of Gln in the polypeptide substrate. Collapse of the thiohemiketal intermediate results in cleavage of the peptide bond, release of the C-terminal part of the polypeptide substrate, and thioester linkage formation between Cys145 and the N-terminal part of the polypeptide substrate. Finally, a water molecule hydrolyzes the thioester linkage and displaces Cys145 to release the N-terminal segment of the polypeptide substrate. Thioester linkage formation is an essential step in the catalytic mechanism of 3CLpro and a target of antiviral development (30).

In addition to the active site, targeting the dimeric interface of SARS-CoV-2 3CLpro is a promising strategy for the structure-based design of drugs against SARS-CoV-2 and other coronaviruses. In the present study, nine residues in the dimer interface, S1, R4, S10, K12, E14, S139, E166, E290, and Q299, were selected for site-directed mutagenesis based on structural analysis, and their impacts on the dimerization and catalytic activity of SARS-CoV-2 3CLpro were evaluated. The findings provide a foundation for the design of unique and effective antivirals targeting coronavirus-based infections.

## Results

### Structural analysis of the dimer interface of 3CLpro SARS-CoV-2

The high degree of structural conservation of 3CLpro among coronaviruses makes it an important therapeutic target against the current SARS-CoV-2 and future coronaviruses. Structure-based drug design can be used to screen and develop inhibitors that either target the enzyme active site to block peptide substrate binding or target its dimerization, which has been proposed to be important for the catalytic activity of 3CLpro. For the latter, identifying residues of the dimer interface that are important for the activity of SARS-CoV-2 3CLpro is essential for effective computational drug screening and further development of antiviral therapeutics against COVID-19.

The previously reported structure of SARS-CoV-2 3CLpro without bound substrate or inhibitor determined at 1.85 Å resolution (Protein Data Bank code: 6WTM) was used for structural analysis of the dimer interface to identify key residues for mutagenesis (31). Three important areas of the dimer interface were identified, as shown in Figure 1. First, the side chain of S1 on the N-finger of one monomer interacts with E166 on domain II of the other monomer, creating two interaction points between the two monomers (Fig. 1, *A* and *B*). Specifically, the side chain of E166 forms ionic interactions at 2.4 Å with the free α-amine group of the N terminus and H-bonding interactions at 3.6 Å with the side chain of S1. Second, the two N-fingers of the dimer interact with one another to create a single point of contact at the dimer interface (Fig. 1*C*). Multiple H-bonding and ionic interactions are formed between the side and main chain atoms of S10, G11, K12, and E14, which are located in a one-turn α-helix at the end of the N-finger. The side chains of S10 from both monomers form 4.0 Å H-bonding interactions, whereas the side chain of E14 forms a 2.8 Å tight H-bond with the amide nitrogen of G11 and long-distance interactions with the side chain of K12 of each monomer at 4.5 and 7.0 Å. Third, the side chain of R4 of the N-finger of one monomer forms a salt bridge at 3.4 Å or 3.6 Å with E290 of domain III of the other monomer, again creating two contact points between the two monomers (Fig. 1*D*). These salt bridge interactions are located in the middle of the dimer interface and are accompanied by tight H-bonding interactions at 2.9 and 3.1 Å between the side chains of S139 and Q299 of domains II and III, respectively. S139 is also part of the oxyanion hole formed by hydrogen bond donors of the main chain amides of the S139–L141 loop in the S1 subsite, which stabilizes formation of the oxyanion transition state during catalysis (11, 25, 26, 27, 28, 29). Based on this structural analysis, S1, R4, S10, K12, E14, S139, E166, E290, and Q299 were selected for mutagenesis to characterize their roles in the activity and dimerization of SARS-CoV-2 3CLpro.

### Oligomeric states of the dimer interface mutants of 3CLpro

**Figure 2:**
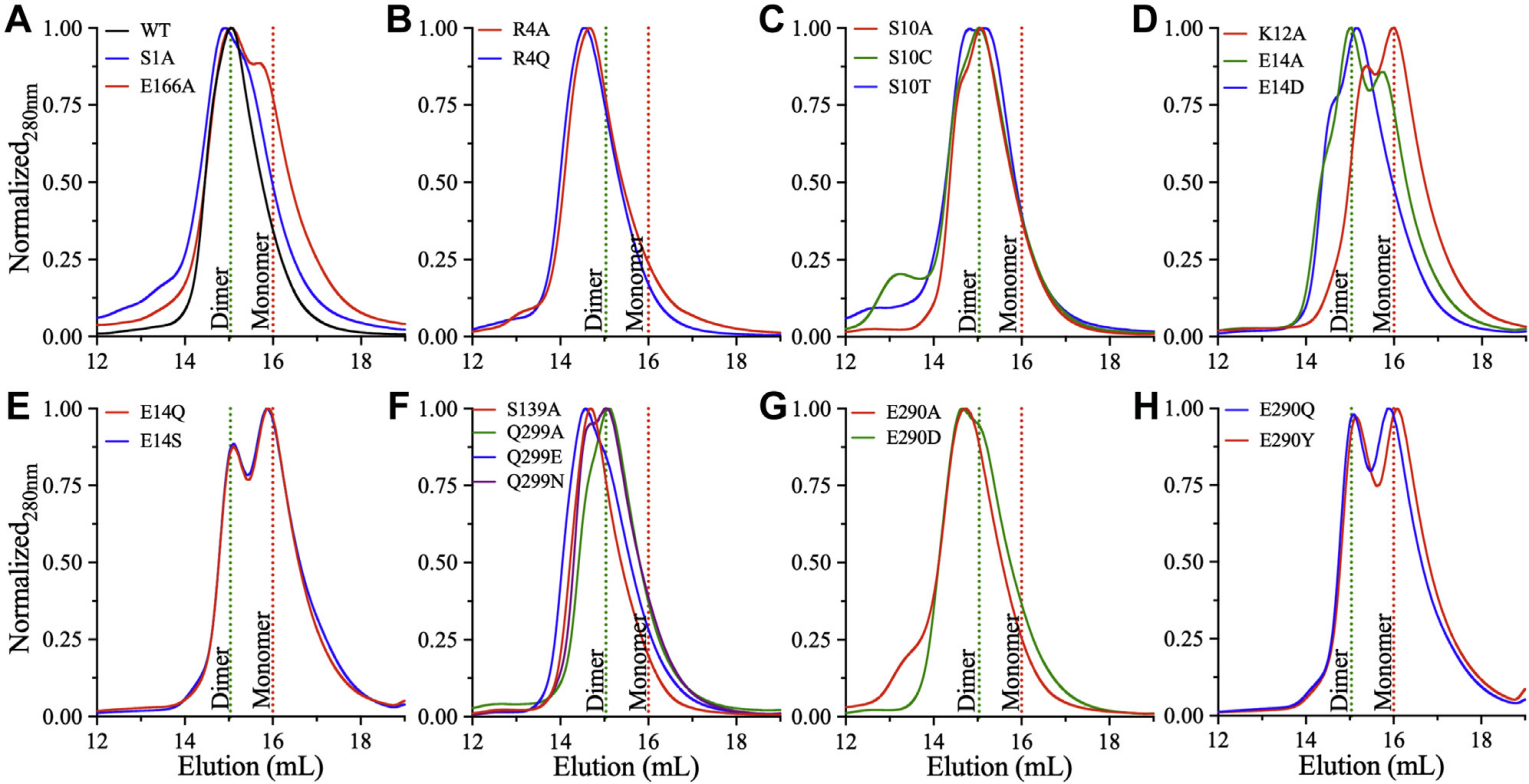
**Analytical size-exclusion chromatography (aSEC) of WT and dimer interface mutants of 3CLpro.***A*, gel filtration profiles (Superdex 200 Increase 10/300 GL; Cytiva Life Sciences/Biacore) of WT (*black*), S1A (*blue*), and E166A (*red*) indicating dimer formation. A shoulder corresponding to the monomer is evident for E166A. *B*, aSEC profiles of Arg4 substitutions. R4A (*red*) and R4Q (*blue*) were mostly dimer. *C*, aSEC profiles of S10A (*red*), S10C (*green*), and S10T (*blue*) indicating dimer formation with retention times similar to that of WT. *D*, gel filtration profiles of mutants in the one-turn α-helix of the N-finger. E14D (*blue*) was a dimer. K12A (*red*) and E14A (*green*) showed an equilibrium between two states with a preference for the monomer or dimer, respectively. *E*, aSEC profiles of E14Q (*red*) and E14S (*blue*) showing an equilibrium between monomeric and dimeric states with a preference for the monomer. *F*, aSEC profiles of S139A (*red*), Q299A (*green*), Q299E (*blue*), and Q299N (*purple*) indicating dimer formation. The retention volume of Q299A/N was similar to that of WT. By contrast, a major peak with a smaller retention volume than WT was observed for S139A or Q299E. *G*, gel filtration profiles of E290A (*red*) and E290D (*green*) indicating dimer formation with a smaller retention volume of the major peak compared with WT. *H*, aSEC profiles of E290Q (*blue*) and E290Y (*purple*) exhibited an equilibrium between the dimer and monomer. The *vertical dashed lines* on all panels are retention volumes of dimeric (*green*) and monomeric (*red*) forms of the WT enzyme, which have molecular weights of 34.5 and 69 kDa, respectively. The enzyme concentration for all runs was >7 mg/ml in 20 mM Hepes buffer, pH 7.0, with temperature of 4 °C. Data are representative of triplicate runs. 3CLpro, 3C-like protease.

To assess the impact of the selected dimer interface residues of SARS-CoV-2 3CLpro on dimerization and activity, single amino acid substitutions were introduced. The mutants were expressed in *Escherichia coli* and purified as previously reported for WT recombinant 3CLpro (32). The oligomeric state of each purified protein was determined by analytical size-exclusion chromatography (aSEC). A minimum enzyme concentration of 7 mg/ml in 20 mM Hepes buffer (pH 7.0) was used for all mutants and WT enzyme at 4 °C, and the retention volumes were compared to assess perturbation of the dimer–monomer equilibrium (Fig. 2). Similar to the WT enzyme, a single early peak corresponding to the dimer (71.6 kDa) was observed for S1A (Fig. 2*A*), S10A/C/T (Fig. 2*C*), E14D (Fig. 2*D*), and Q299A (Fig. 2*F*). The other mutants exhibited both an early peak and a late peak, indicating an equilibrium between the dimer and monomer (35.8 kDa), including E166A (Fig. 2*A*), K12A (Fig. 2*D*), E14A/Q/S (Fig. 2, *D* and *E*), and E290Q/Y (Fig. 2*H* and Table S1). For R4A/Q, a single peak was observed with a smaller retention volume than that for the WT enzyme (Fig. 2*B*). This earlier peak indicates that R4A/Q adopts a different shape or an oligomerization state that is larger than the dimeric state of the WT enzyme. In addition, two peaks were observed for S139A, E290A/D, and Q299E; the retention volume of the second peak was similar to that of the WT enzyme (Fig. 2, *F* and *H*). The R4A/Q, S139A, E290A/D, and Q299E mutants were the only variants with a peak with a smaller retention volume than the WT enzyme.

### Thermodynamic stability of the dimer interface mutants of 3CLpro

**Figure 3:**
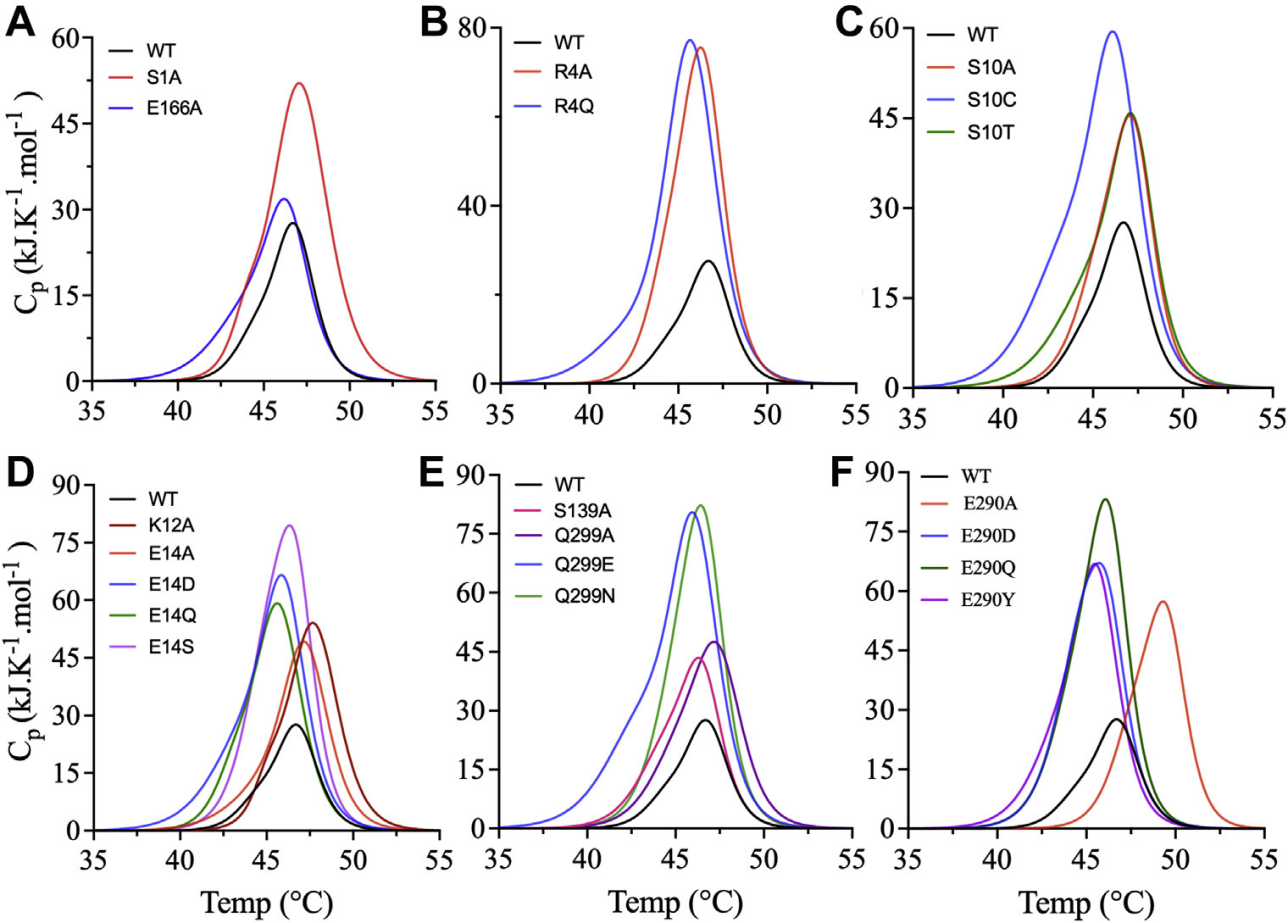
**Effects of dimer interface mutations on the thermodynamic stability of 3CLpro.***A*–*F*, DSC thermograms of the dimer interface mutants of 3CLpro. All enzymes exhibited a single transition with an early shoulder peak. For all mutants, the thermogram amplitude was higher than that for WT. Data were confirmed by triplicate runs. 3CLpro, 3C-like protease; DSC, differential scanning calorimetry.

The effects of the dimer interface mutations on overall thermodynamic stability were assessed by differential scanning calorimetry (DSC). The DSC thermograms of WT and mutant 3CLpro (Fig. 3) were acquired in 20 mM Hepes (pH 7.0), 150 mM NaCl, and 20% (v/v) dimethyl sulfoxide (DMSO), similar to the buffer subsequently used to assay catalytic activity. The temperature was ramped from 15 to 75 °C at a scan rate of 1 °C/min to acquire the thermal unfolding transitions. The *T*_m_ was calculated at the apex of the melting peak, and the calorimetric enthalpy (*ΔH*_cal_) was determined from the area under the thermographic peak. The WT enzyme exhibited a single transition with an early shoulder peak (Fig. 3*A*). For all mutants, the overall shape of the thermogram was similar to that of WT, with a single thermal transition (Fig. 3,*A*−F). 3CLpro E290A exhibited the highest *T*_m_, 49.5 ± 0.2 °C, compared with 46.7 ± 0.1 °C for WT. The *T*_m_ values of the other mutants were close to that of WT (Fig. S1, *A* and *B*). Compared with WT, the majority of the dimer interface mutants exhibited significantly higher amplitudes of the DSC thermographic transition (Fig. 3,*A*−*F*). For R4Q, S10C, E14Q, E14S, E290Q, and Q299N, *ΔH*_cal_ increased by threefold compared with WT (133 ± 4 kJ/mol), with an average value of ∼350 kJ/mol (Fig. S1, *C* and *D*).

### Relative activity of the dimer interface mutants

**Figure 4:**
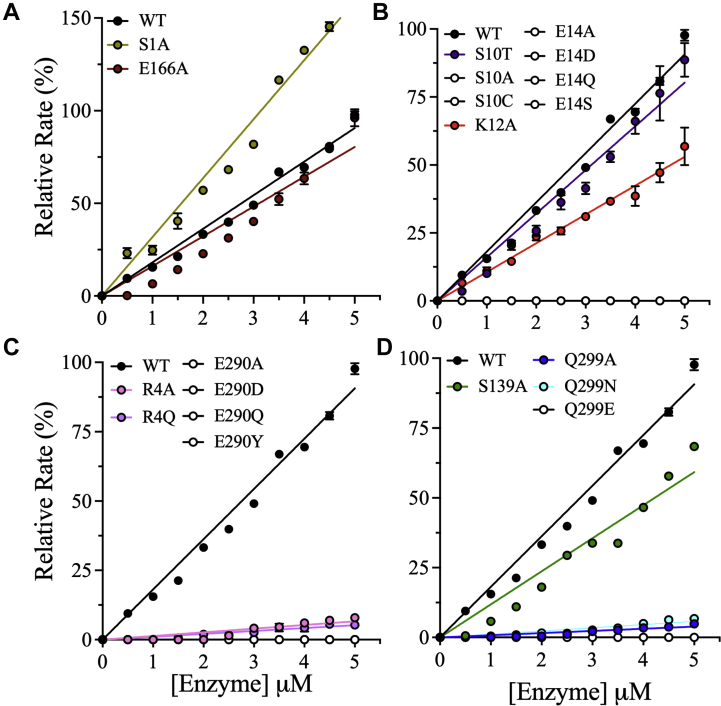
**Effects of dimer interface mutations on the relative activity of 3CLpro.***A*–*D*, the relative enzymatic activities of the dimer interface mutants of 3CLpro were measured at different enzyme concentrations up to 5.0 μM. The assays were performed in 20 mM Hepes (pH 7.0), 150 mM NaCl, 1 mM EDTA, 1 mM TCEP, and 20% (v/v) DMSO at a fixed peptide substrate concentration of 60 μM. The relative rate was calculated by normalization to the rate of WT, which was set to 100%. The data for mutants with no enzymatic activity are shown as *open black circles*. The data for enzymatically active mutants are color coded, and the data for WT are shown as *filled black circles*. Data points are means ± SD of triplicate measurements. 3CLpro, 3C-like protease; DMSO, dimethyl sulfoxide; TCEP, Tris(2-carboxyethyl)phosphine.

The proteolytic activity of the dimer interface mutants was measured in 20 mM Hepes (pH 7.0), 150 mM NaCl, 1 mM EDTA, 1 mM Tris(2-carboxyethyl)phosphine (TCEP), and 20% (v/v) DMSO at a fixed concentration of the fluorescent peptide substrate of 60 μM as described previously (32, 33, 34). This substrate concentration is equal to the *K*_*m*_ of the WT enzyme, and the addition of 20% DMSO maximizes activity by reducing aggregation of the peptide substrate. The WT and mutant enzyme concentrations were varied from 0.5 to 5.0 μM, and the relative rate of each mutant was calculated by comparing the slope of the straight line of the plot of activity as a function of enzyme concentration to that of the WT enzyme (Fig. 4,*A*−*D*). Interestingly, mutation to alanine of the residues at the first site of interest in the dimer interface, S1 and E166 (Fig. 1, *A* and *B*), twofold enhancement, or reduced proteolytic activity to 89% of the WT enzyme, respectively (Figs. 4*A* and S2*A*). The relative activity of 3CLPro S1A was double that of WT, whereas E166A exhibited 89% relative activity.

At the second dimer interface site of interest (Fig. 1*C*), introduction of S10A or E14A eliminated 3CLpro proteolytic activity (Fig. 4*B*). The role of S10 was further investigated by introducing S10C or S10T. Like S10A, 3CLPro S10C was inactive; by contrast, the activity of 3CLPro S10T was 90% of the WT activity (Figs. 4*B* and S2*A*). The inactivation of 3CLpro by E14A was further explored by examining the bonding interactions of E14 with the side chain of K12 (Fig. 1*C*). The introduction of K12A reduced the activity of 3CLpro by 40% compared with WT (Fig. S2*A*). Alternative amino acid substitutions at E14, that is, E14D, E14Q, and E14S, did not recover the activity of 3CLpro (Fig. 4*B*).

Mutation of residues at the third dimer interface site of interest (Fig. 1*D*) greatly reduced 3CLpro activity. In particular, introduction of alanine at R4 or E290, which form ionic pairs at two contact points between the monomers, reduced relative activity to 16% or eliminated activity, respectively (Fig. 4*C*). Among alternative amino acid substitutions, 3CLPro R4Q had a relative activity of only 6%. All amino acid substitutions of E290, including E290D, E290Q, and E290Y, resulted in a lack of detectable proteolytic activity. In the same dimer interface pocket, S139 forms H-bonding interactions with Q299 (Fig. 1*D*), and 3CLpro S139A, Q299A, and Q299N exhibited relative activities of 46%, 4%, and 6%, respectively (Fig. 4*D*).

### Kinetic characterization of the active dimer interface mutants

**Figure 5:**
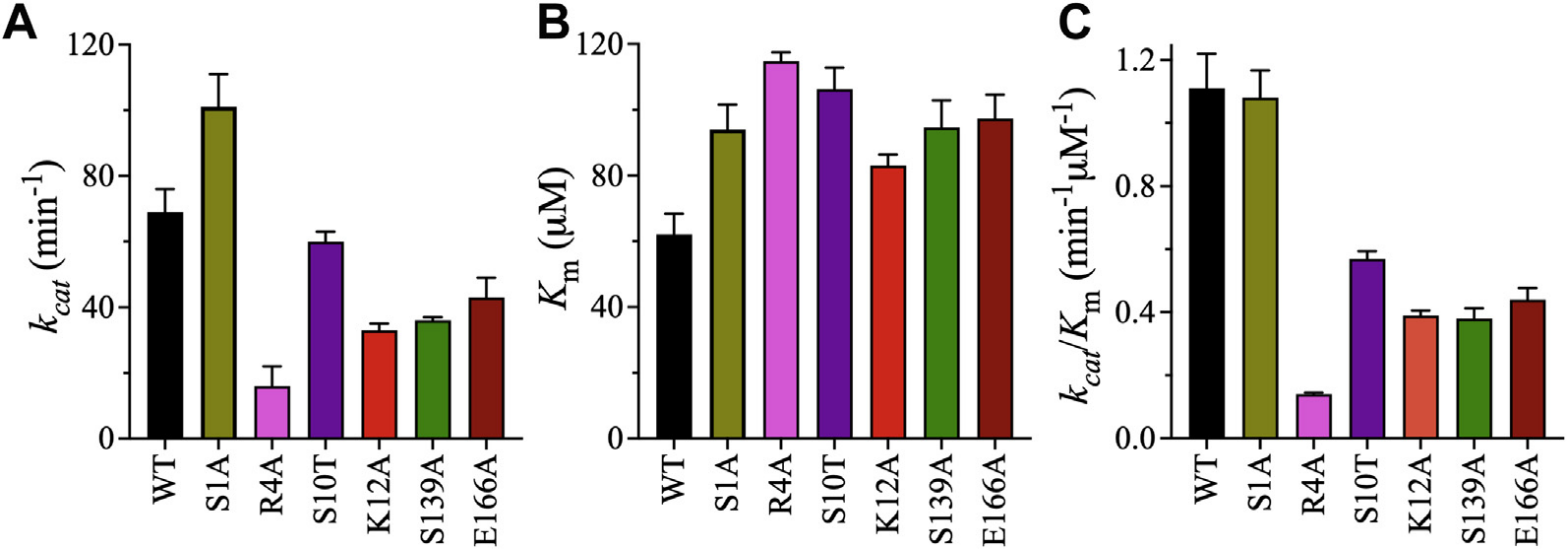
**Effects of dimer interface mutations on the kinetic parameters of 3CLpro.***A* and *B*, bar plots of *k*_cat_ and *K*_*m*_ values of WT and dimer interface mutants of 3CLpro. All mutants exhibited decreased *k*_cat_ except S1A, which had higher *k*_cat_ than WT. In addition, all mutants had reduced affinity for the peptide substrate, as indicated by increases in their *K*_*m*_ values compared with WT. *C*, bar plot of the catalytic efficiency (*k*_cat_/*K*_*m*_) of the dimer interface mutants of 3CLpro. R4A had the lowest catalytic efficiency, whereas the catalytic efficiency of S1A was equivalent to that of WT. Thus, these residues have different impacts on the activity of 3CLpro despite their proximity in the N-finger. Data points are means ± SD of triplicate measurements. 3CLpro, 3C-like protease.

To compare the kinetic parameters of the active mutants of 3CLpro with the WT enzyme, initial velocity studies were performed at 30 °C in 20 mM Hepes (pH 7.0), 150 mM NaCl, 1 mM EDTA, 1 mM TCEP, and 20% (v/v) DMSO. The S1A mutation increased *k*_cat_ by ∼150% compared with WT, whereas all other mutations decreased *k*_cat_ (Fig. 5*A*). The reductions of *k*_cat_ ranged from twofold for K12A and S139A to fourfold for R4A (Table S2), whereas S10T and E166A had smaller impacts on the *k*_cat_ of 3CLpro. All mutations increased *K*_*m*_ of 3CLpro by less than twofold compared with WT (62 ± 6 μM); 3CLpro R4A had the lowest affinity for the peptide substrate (Fig. 5*B* and Table S2). Thus, the effects of the dimer interface mutants on *k*_cat_ were more pronounced than their effects on *K*_*m*_. Overall, the catalytic efficiency (*k*_cat_/*K*_*m*_) of S1A was unchanged compared with WT, whereas all other mutants exhibited reductions of catalytic efficiency (Fig. 5*C*). The R4A mutant exhibited the largest decrease in catalytic efficiency, sevenfold, whereas the S10T, K12A, S139A, and E166A mutants displayed reductions of twofold to threefold.

## Discussion

In the fight against COVID-19 and the spread of SARS-CoV-2, the discovery of antiviral drugs and the development of therapeutics are of great importance. A conserved step in the maturation of coronaviruses is the processing of the replicase polyproteins to produce new virus particles. The 3CLpro protease is responsible for replicase polyprotein processing and the release of functional proteins during virus infection, making it an attractive target for the development of antiviral therapeutics against COVID-19. In this study, we evaluated the importance of dimer interface residues for the catalytic activity of SARS-CoV-2 3CLpro to support the screening and design of antivirals. Dimerization is thought to be essential for the activity of SARS-CoV-2 3CLpro, likely by ensuring the correct conformation of the Gly138–Leu141 loop that forms the oxyanion hole (13, 35). Small molecules that target the dimer interface rather than the active site will not act as competitive inhibitors of 3CLpro, ensuring that inhibition cannot be overcome by increasing the substrate concentration. Such inhibitors would make more effective antivirals than molecules that bind the active site. In this study, three sites in the dimer interface were identified as potentially important for the dimerization of 3CLpro (Fig. 1) and were targeted for mutagenesis to evaluate their roles in protease dimerization and activity.

### Site 1: S1 and E166

S1 and E166 are expected to form H-bonding and ionic interactions at two points of contact between the two monomers of SARS-CoV-2 3CLpro. Specifically, the side chain of E166 forms H-bonding interactions with the side chain of S1 and tight ionic interactions with the α-amine of the N terminus (Fig. 1, *A* and *B*). Interestingly, introduction of S1A increased the relative activity and catalytic turnover (*k*_cat_) of 3CLpro compared with WT; in SARS-CoV 3CLpro, the same mutation reduced relative activity by 54% compared with WT (23). The dimerization and catalytic efficiency (*k*_cat_/*K*_*m*_) of S1A were similar to those of WT SARS-CoV-2 3CLpro; similar results were obtained previously for mutation of this residue in SARS-CoV 3CLpro (23). These results suggest that the interactions of S1 are not essential for the dimerization or activity of 3CLpro. Moreover, the finding that S1A increased activity has important implications for the virtual screening and design of small molecules that inhibit the activity of 3CLpro by binding at the dimer interface. Disrupting the interactions of S1 may be undesirable, as doing so may increase the activity of SARS-CoV-2.

In SARS-CoV 3CLpro, E166 is thought to act as a link between the substrate-binding site and the dimer interface (36), and introduction of E166A in this enzyme decreased *k*_*cat*_ by twofold compared with WT and eliminated the substrate-induced dimerization of the R298A mutant. In Middle East respiratory syndrome coronavirus 3CLpro, which exhibits weak dimerization that is enhanced in the presence of substrate, introduction of an alanine at this residue (E169) reduced *k*_cat_ by appoximately sixfold and resulted in a shift to the monomer form even in the presence of substrate (37). In the present study, introduction of E166A retained 89% of the WT proteolytic activity and had little effect on the dimer:monomer equilibrium. In addition to their side-chain interaction, Ser1 and Glu166 are in close vicinity to Phe140, His163, and His172, key residues involved in the binding of peptide substrate (38). Molecular dynamics (MD) simulations on 3CLpro from both SARS-CoV-2/CoV confirmed that the protonation state of His172 and its interaction with Phe140, His163, and Glu166 are important in shaping the S1 substrate-binding pocket of 3CLpro (39, 40). Virtual and X-ray–based screens have suggested a role of E166 in noncovalent interactions with potential inhibitors of SARS-CoV-2 3CLpro (30, 41), but this study is the first to evaluate the impact of this residue on activity and dimerization. In summary, the results of the present study suggest that the interactions of S1 and E166, whether with each other, other residues or substrate, are not major contributors for the dimerization or activity of 3CLpro.

### Site 2: S10, K12, and E14

The second site of interest at the dimer interface comprises residues S10, K12, and E14, which are located in a one-turn α-helix at the end of the N-finger; the two monomers interact with one another at this site to form a single contact point at the dimer interface (Fig. 1*C*). Similar interactions have been proposed for SARS-CoV 3CLpro (42). In SARS-CoV 3CLpro, introduction of S10A eliminated activity and reduced the share of the dimer form by ∼20% (23). In the present study, SARS-CoV-2 3CLpro S10A was also inactive, as was S10C, which is unable to facilitate H-bonding interactions between the two monomers. By contrast, S10T was fully active, despite the larger size of the threonine side chain compared with serine. Thus, the H-bonding interactions mediated by the side chain of S10 in both monomers, not the size of the side chain, are important for the catalytic activity of 3CLpro. Unlike SARS-CoV 3CLpro S10A, the S10 mutants of SARS-CoV-2 3CLpro were largely present as dimers.

The side chain of E14 forms long-range (∼4.5 and 7 Å) ionic interactions with the side chain of K12 but strong H-bonding interactions with the backbone amine nitrogen of Gly11 (Fig. 1*C*). The introduction of K12A reduced the relative activity of 3CLpro by ∼40% (Fig. S2*A*), indicating that the interaction between the side chains of K12 and E14 is important but not crucial for protease activity. However, 3CLpro E14A was catalytically inactive, consistent with an important role of the interaction of the side chain of E14 with the backbone of Gly11 for 3CLpro activity. Further support for this role is provided by the inactivity of 3CLpro E14Q, despite the similar molecular sizes of glutamine and glutamate. Moreover, 3CLpro E14D and E14S were also inactive, suggesting that the distance between the N-fingers of the monomers and the polarity of the side chain of E14 are important for catalytic activity. Previous mutagenesis studies have examined only E14A in SARS-CoV 3CLpro; this mutant exhibited a decrease in the dimer form of ∼50% and 4% relative activity compared with WT (23).

The analysis of the oligomerization of these mutants emphasized that dimerization of 3CLpro is not sufficient for protease activity, even when the active site is intact (Fig. S2, *A* and *B*). Like WT, the active mutant S10T and the inactive mutants S10A/C and E14A/D/Q/S were largely present as dimers. By contrast, the monomer form was more dominant at 60% for the reduced activity of mutant K12A (Table S1), and the inactive mutant E14A also exhibited a shoulder on aSEC corresponding to the monomer form. The reduced activity of 3CLpro K12A compared with the WT enzyme may be attributable to this increase in the monomer form. Regardless of activity or oligomeric state, all mutations in the N-finger of 3CLpro increased the *ΔH*_cal_ of the protein as measured by DSC, with only small changes in *T*_m_ (Fig. S1, *A*–*D*). These increases in *ΔH*_cal_ are attributable to changes in the overall bonding characteristics of the protein and indicate enhanced polar bonding interactions and hydrophilicity (43, 44, 45). Overall, the analyses of mutations at this site suggest that the interactions initiated by S10 or E14 are critical for the activity of 3CLpro but not its dimerization.

### Site 3: R4, S139, E290, and Q299

The third site of interest in the dimer interface includes the salt bridge between the side chains of R4 and E290 of the N-finger and domain III, respectively, and the H-bonding interactions between the side chains of S139 and Q299 of domains II and III, respectively (Fig. 1*D*). The four residues of the third site create two contact points between the monomers that enhance the stability of the dimeric state. These residues are also conserved in SARS-CoV 3CLpro and participate in similar interactions (42, 46).

An important role of R4 in coronavirus 3CLpro was first identified in mutagenesis studies targeting the N and C termini of SARS-CoV 3CLpro; removal of residues 1 to 3 from the N terminus had no effect on dimerization and reduced relative activity by 24%, whereas removal of residues 1 to 4 largely eliminated both activity and dimerization (19). Similarly, Chen *et al.* (23) found that SARS-CoV 3CLpro R4A exhibited 10% relative activity and a 20% decrease in dimerization. However, Chou *et al.* (47) observed that mutation of R4 to alanine had very little effect on activity but decreased dimerization by 80%. In the present study, SARS-CoV-2 3CLpro R4A and R4Q exhibited 16% and 6% relative activity, respectively, with both mutants mostly dimeric with a smaller retention volume on aSEC than the WT enzyme (Fig. 2*B*). Even though S1 and R4 are both part of the long loop at the N terminus and interact with domains II and III of the other monomer, respectively (Fig. 1, *A* and *D*), mutation of these residues to alanine had very different effects on the catalytic activity of 3CLpro: S1A and R4A had relative activities of 200% and 16%, respectively (Fig. S2, *A* and *B*).

The importance of R4 in maintaining 3CLpro activity is most likely because of its interactions with E290, as all mutations of the latter residue eliminated protease activity. Like the R4 mutants, the E290 mutants had diverse oligomeric states, with larger retention volumes for E290A/D and smaller retention volumes for E290Q/Y compared with the WT enzyme. Introduction of E290A was previously reported to eliminate activity for SARS-CoV 3CLpro (47). Even though are present as dimers, the lack of catalytic capabilities of R4A/Q and E290A/D mutants can be a result of formation of a new type of dimer that has been shown previously for 3CLpro of SARS-CoV to be catalytically inactive (22). The low proteolytic activity in the presence of R4A mutant was also observed in 3CLpro from porcine epidemic diarrhea virus, where dimeric state of the protease was maintained (16).

In addition to its interactions with Glu290, Arg4 interacts at 2.8 Å with the backbone carbonyl oxygen of Lys137 of the oxyanion L1 loop (residues 138–145) (17, 48). These key interactions shape the S1 substrate-binding pocket in addition to His163 and Glu166 that assist in binding the peptide substrates and Phe140 and His172 in maintaining the open state of 3CLpro (17, 36, 48). MD simulation also revealed the importance of residues Gly143, Ser144, and Cys145 of the L1 loop in the inhibition of 3CLpro (49). The L1 loop has been shown to be a promising target to improve the design of selective and efficient inhibitors targeting the 3CLpro of SARS-CoV-2 (49).

Among the other residues in the R4/E290 pocket, Q299 was more important for the catalytic activity of SARS-CoV-2 3CLpro than S139; S139A exhibited 46% relative activity, whereas only residual activity of Q299A/N was observed (Fig. S2, *A* and *B*). Consistent with these findings, in a previous study, the Q299A and Q299N mutants of SARS-CoV 3CLpro exhibited reductions of *k*_cat_ of ∼45-fold and ∼19-fold, respectively, whereas S139A exhibited no reduction of activity or dimerization compared with WT (46). MD simulations of ligand complexes of SARS-CoV-2 3CLpro suggest that a water-mediated interaction between S139 of one monomer and Q299 of the other monomer links dimerization with appropriate active-site conformation, as S139 is also part of the oxyanion hole (50).

Like the portion of the N-finger (site 2) described previously, no clear relationship between oligomeric state and activity was observed for mutations in this third site of the dimer interface of 3CLPro. The residually active mutants Q299A/N and the inactive mutant Q299E were all present in dimer form, although the retention volumes of Q299E/N were larger than that of the WT enzyme (Figs. 2*F* and S2*B*). However, R4A and R4Q, which exhibited similarly low levels of residual activity, were present in primarily dimeric form (Fig. 3*B*). Moreover, the inactive mutants E290A/D were present as dimers, but the similarly inactive mutants E290Q/Y exhibited a shift toward the monomer state. DSC revealed that among all mutations at all sites, the inactive mutant E290A had the highest *T*_m_, 49.5 ± 0.2 °C (Fig. S1*B*).

## Conclusion

The introduction of mutations of key residues of the dimer interface of SARS-CoV-2 3CLpro confirmed the importance of some of these residues for dimerization and activity. All the 3CLpro mutants with at least 50% relative activity, with the exception of K12A, exhibited an oligomer state similar to that of the dimeric WT enzyme, including S1A, S10T, S139A, and E166A. The K12A mutant exhibited an equilibrium between the two states, with a preference for the monomer, and 60% relative activity compared with WT. By contrast, the oligomeric states of the inactive mutants spanned a wide range, with aSEC retention volumes that were similar, larger, or smaller than that of the WT enzyme. The results presented here demonstrate that dimerization does not guarantee a catalytically active 3CLpro, where some mutants that formed dimers were inactive. As has been shown previously for SARS-CoV 3CLpro (22), the dimeric form of catalytically inactive mutants can be a result of formation of new types of dimers that are different from that of the WT enzyme. There was also no clear link between thermodynamic stability and activity: S10T, which was as active as WT, had *T*_m_ and *ΔH*_cal_ values similar to those of WT, whereas S1A, which had higher activity than the WT enzyme, exhibited a twofold increase in *ΔH*_cal_ compared with WT (Fig. 3*G*).

This study is one of the first to directly probe the roles of dimer interface residues of SARS-CoV-2 3CLpro through mutagenesis, biophysical characterization, and activity assays. A number of studies have performed virtual or high-throughput structural biology–based screens of potential inhibitors of 3CLpro (41, 51, 52), but the findings have not been complemented by biochemical assessments of the roles of specific residues or the effects of proposed inhibitors. The computational screening of inhibitors that bind the active site of 3CLpro will more likely yield competitive inhibitors that are less potent than noncompetitive inhibitors targeting allosteric sites in the dimer interface. The work described here will help direct computational screening efforts to develop effective inhibitors that bind the dimer interface.

## Experimental procedures

### Expression and purification of 3CLpro variants

Recombinant 3CLpro genes encoding the WT or mutant forms of the enzyme were introduced into the pET28b(+) bacterial expression vector by GenScript, Inc. Hisx6-tagged human 3CLpro protein was expressed in *E. coli* BL21-CodonPlus-RIL (Stratagene). The inoculated culture (2–6 l) was grown in terrific broth medium at 30 °C in the presence of 100 mg/l kanamycin and 50 mg/l chloramphenicol until the absorbance reached 0.8 at 600 nm. The temperature was then lowered to 15 °C, and expression was induced overnight with 0.5 mM IPTG. The cells were harvested by centrifugation at 12,000*g* at 4 °C for 10 min in an Avanti J26-XPI centrifuge (Beckman Coulter, Inc) and resuspended in lysis buffer (20 mM Tris [pH 7.8], 150 mM NaCl, 5 mM imidazole, 3 mM β-mercaptoethanol [β-ME], and 0.1% protease inhibitor cocktail from Sigma–Aldrich: P8849). The cells were lysed by sonication on ice and centrifuged at 40,000*g* for 45 min at 4 °C. The supernatant was loaded on a ProBond Nickel-Chelating Resin (Life Technologies) previously equilibrated with binding buffer (20 mM Tris [pH 7.5], 150 mM NaCl, 5 mM imidazole, and 3 mM β-ME) at 4 °C. The resin was washed with 10 column volumes of binding buffer, followed by 15 column volumes of washing buffer (20 mM Tris [pH 7.5], 150 mM NaCl, 25 mM imidazole, and 3 mM β-ME). The His-tagged 3CLpro enzyme was eluted from the column in 20 mM Tris, pH 7.5, 150 mM NaCl, 300 mM imidazole, and 3 mM βME and collected in aliquots of 1 ml. Finally, the nickel-column fractions containing 3CLpro were loaded onto a HiLoad Superdex 200 size-exclusion column (GE Healthcare) using an AKTA purifier core system (GE Healthcare). The column was pre-equilibrated with filtration buffer (20 mM Hepes [pH 7.5], 150 mM NaCl, and 0.5 mM TCEP). The final protein was collected and concentrated to ∼150 μM based on the Bradford assay, and the sample purity was assessed *via* SDS-PAGE (Fig. 2*A*).

### aSEC analyses

The oligomeric states of the 3CLpro mutants were analyzed by aSEC on a Superdex 200 Increase 10/300 GL using an AKTA pure protein purification system (Cytiva Life Sciences). The column was pre-equilibrated with 20 mM Hepes (pH 7.5), and 50 μl of protein sample with a concentration of >7 mg/ml was injected at a flow rate of 0.75 ml/min and temperature of 4 °C. The 4 °C temperature is important to maintain 3CLpro in the dimeric state as was shown previously for 3CLpro SARS-CoV that room temperature induces an equilibrium between the monomeric and dimeric states of the enzyme (22). Each variant was analyzed three times to confirm the reliability of the data. The absorbance signal was normalized to the maximum value recorded at 280 nm for the different mutants of 3CLpro. Molecular weight standards were used to calibrate the column using a low molecular mass gel filtration kit (Cytiva Life Sciences/Biacore) with carbonic anhydrase (29.6 kDa) and ribokinase (70 kDa) as marker proteins mimicking the monomeric and dimeric forms of the WT enzyme, which have molecular weights of 34.5 and 69 kDa, respectively.

### DSC

The thermodynamic stability of 3CLpro was measured using a Nano DSC instrument (TA Instruments) calibrated using chicken egg white lysozyme, a known external Nano DSC standard that is part of the TA Instruments test kit (602198.901). The thermogram was acquired at a 3CLpro concentration of 30 μM in 20 mM Hepes (pH 7.0), 150 mM NaCl, and 20% (v/v) DMSO. The protein samples were heated from 15 to 75 °C at a scan rate of 1 °C/min and 3 atm pressure. The background scans were obtained by loading degassed buffer in both the reference and sample cells and heating at the same rate. The DSC thermograms were corrected by subtracting the corresponding buffer baseline and converted to plots of excess heat capacity (*C*_p_) as a function of temperature. The *T*_m_ was determined at the maximum temperature of the thermal transition, and the *ΔH*_cal_ of the transition was estimated from the area under the thermal transition using Nano Analyzer software (TA Instruments).

### Enzymatic activity analysis and initial velocity studies

The catalytic activities of WT and mutant 3CLpro were measured by a FRET-based assay using the 14-amino-acid fluorogenic peptide substrate (DABCYL)KTSAVLQ↓SGFRKME(EDANS)-NH_2_ (GenScript, Inc) as described previously (10, 53, 54, 55, 56). The reaction was initiated by adding 3CLpro to the peptide substrate in 20 mM Hepes, pH 7.0, 150 mM NaCl, 1 mM EDTA, and 1 mM TCEP. The enzyme concentration was fixed at 3 μM, and the reaction rate was measured for 5 min at 30 °C in a thermostatically controlled cell compartment. The assay buffer contained 20% (v/v) DMSO to reduce the aggregation of the peptide substrate (34). The catalytic rate was determined from the cleavage of the fluorogenic substrate, which was monitored by the increase in the fluorescence signal upon release of the EDANS group in a 96-well plate assay format in a Cytation 5 multimode microplate reader (Biotek Instruments). The fluorescence signal was monitored at λ_excitation_ of 360 nm and λ_emission_ of 500 nm. To account for the inner filter effect in the FRET enzymatic assay, first, the excitation coefficient of free EDANS was determined in the absence of the peptide substrate by varying the concentration of free EDANS, $f^{0}\left(EDANS\right)$. Next, the correction factor (*Corr%*) required to correct for the decrease in the emission signal of the fluorogenic substrate in the presence of the quencher (DABCYL) was calculated (54, 55, 57, 58). To calculate *Corr%*, the fluorescence of a fixed concentration (50 μM) of free EDANS was measured in the absence, f(S), and presence, f(S+EDANS), of various concentrations of the peptide substrate (from 20 to 500 μM):

$$f^{s}\left(EDANS\right)\;=\;f\left(S\;+\;EDANS\right)\;-\;f\left(S\right)$$

To determine *Corr%,* the emission reduction of free EDANS at a specific substrate concentration, $f^{s}\left(EDANS\right)$, was compared with that of EDANS in the absence of peptide substrate, $f^{0}\left(EDANS\right)$.

$$Corr\;=\;\frac{f^{s}\left(EDANS\right)}{f^{0}\left(EDANS\right)}$$

The calculated *Corr%* at different peptide substrate concentrations was taken into consideration when measuring the cleavage rate of 3CLpro.

To assess the effect of a single amino acid substitution on the rate of proteolysis by 3CLpro, the enzymatic activity of the WT enzyme and dimer interface mutants was first assayed at different enzyme concentrations. The concentration of the peptide substrate was fixed at 60 μM, and the enzyme concentration was varied from 0.5 to 5 μM. Next, initial velocity studies were performed with the WT enzyme and the catalytically active mutants to determine *K*_*m*_ and *k*_cat_. The concentration of the peptide substrate was varied from 20 to 500 μM at a fixed concentration of 3CLpro. The cleavage rate data were fit to the Michaelis–Menten equation using the global fitting analysis function in the kinetics module of SigmaPlot (Systat Software, Inc). Error bars were calculated from triplicate measurements of each reaction, and the results are presented as the mean ± SD.

## Data availability

All relevant data are contained within the article and its supporting information.

## Supporting information

This article contains supporting information.

## Conflict of interest

The authors declare that they have no conflicts of interest with the contents of this article.

## Supporting information

Supporting information_3CLpro Dimer interface
