# Supplementary material for: Key dimer interface residues impact the catalytic activity of 3CLpro, the main protease of SARS-CoV-2
Source: J Biol Chem. 2022 May 11;298(6):102023. doi: 10.1016/j.jbc.2022.102023 (PMC9091064; doi:10.1016/j.jbc.2022.102023)
Supplement: Supporting information_3CLpro Dimer interface [file mmc1.docx]

**Supporting information**

**Key dimer interface residues impact the catalytic activity of 3CLpro, the main protease of SARS-CoV-2**

J**uliana C. Ferreira^1^, Samar Fadl^1^, Wael M. Rabeh^1*^**

^1^Science Division, New York University Abu Dhabi, PO Box 129188, Abu Dhabi, United Arab Emirates


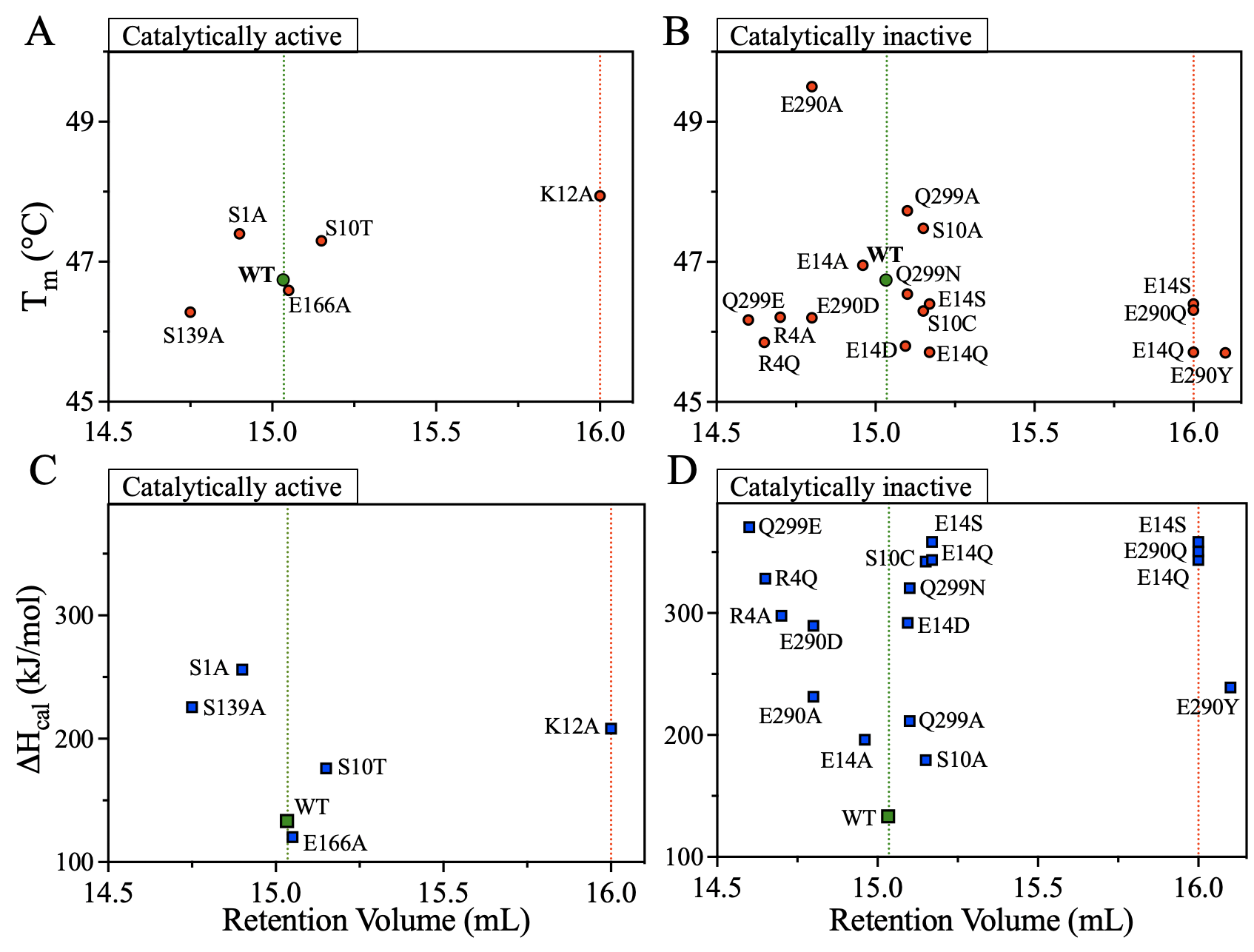


**Figure S1.** **Effects of dimer interface mutations on the thermodynamic stability of 3CLpro**. **(A**-**B)** Plot of the *T*_m_ values (red circles) calculated from the DSC thermal scans of the 3CLpro mutants against the retention volumes from aSEC analysis (Figure 2). The catalytically active and inactive mutants are shown in panels A and B, respectively. The T_m_ value (green circle) of WT 3CLpro is included as controls. **(C**-**D)** Plot of the *ΔH*_cal_ values (blue squares) calculated from the DSC thermal scans of the 3CLpro mutants against the retention volumes from aSEC analysis (Figure 2). The catalytically active and inactive mutants are shown in panels C and D, respectively. The *ΔH*_cal_ value (green squares) of WT 3CLpro is included as controls. The vertical dashed lines on all panels are retention volumes of dimeric (green) and monomeric (red) states of 3CLpro, which have molecular weights of 34.5 and 69 kDa, respectively. The WT enzyme of 3CLpro elutes as a dimer.


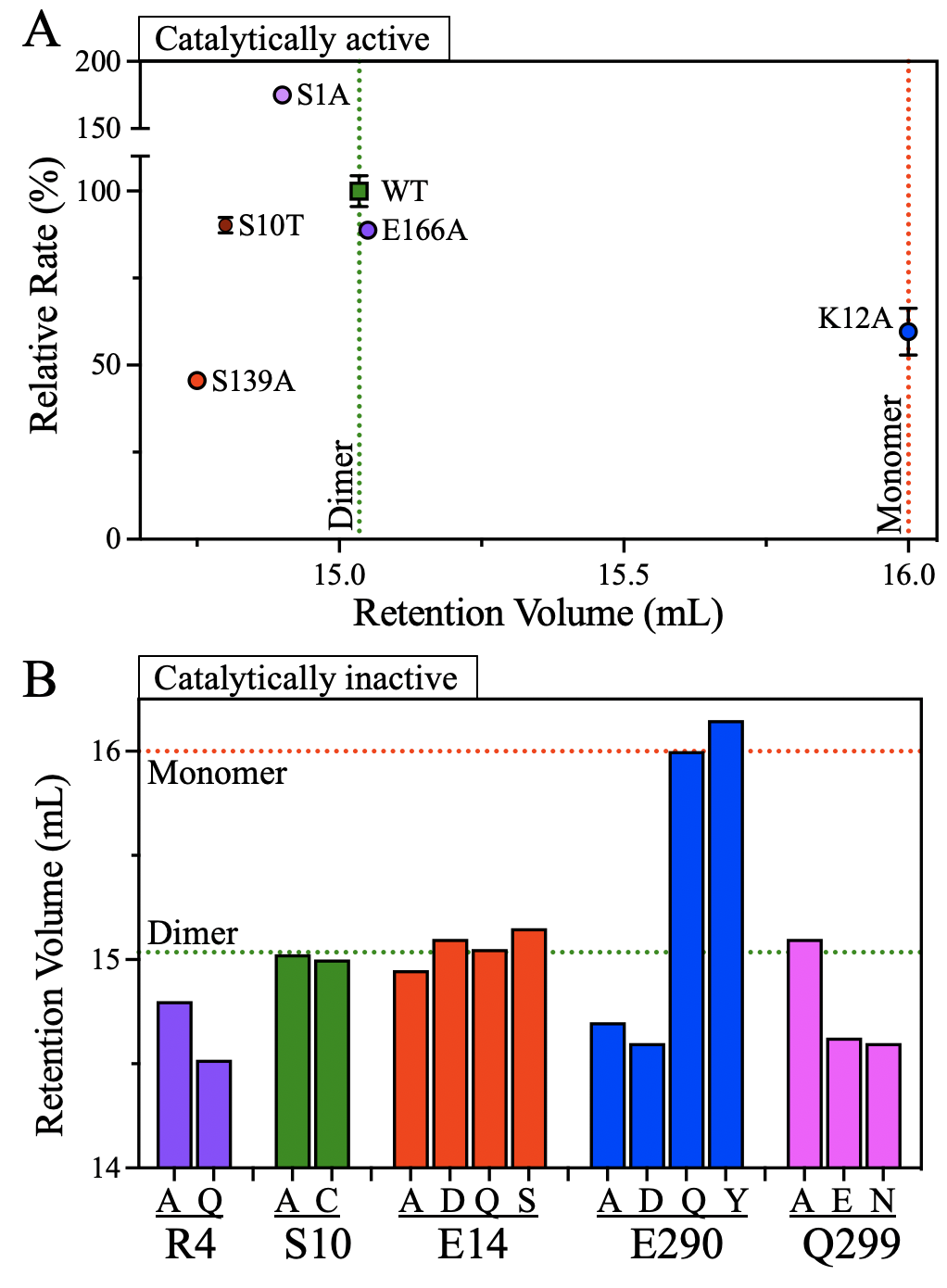


**Figure S2: Effects of dimer interface mutations on the relative activity of** **3CLpro**. (**A**) Plot of the relative activities of the WT enzyme and the catalytically active dimer interface mutants against the retention volumes from aSEC analysis (Fig. 2). The relative rates of the dimer interface mutants decreased with decreasing oligomeric state. (**B**) Bar plot of the retention volumes from aSEC (Fig. 2) of the catalytically inactive dimer interface mutants. The Arg4 (purple) mutants, E290A/D (blue), and Q299E/N (pink) displayed retention volume lower than the dimeric state of the WT enzyme. S10A/C (green), E14A/D/Q/S (red), and Q299A (pink) had retention volumes similar to that of the WT enzyme, indicating that dimer formation does not guarantee catalytically active 3CLpro. The E290Q/Y (blue) displayed the greatest shift toward a monomeric state (largest retention volume).

**Table S1:** Percentage of monomer and dimer from aSEC analysis.

| Mutants | Dimmer | Monomer |
| --- | --- | --- |
| K12A | 40% | 60% |
| E14A | 53% | 47% |
| E14Q | 35% | 65% |
| E14S | 33% | 67% |
| E166A | 68% | 32% |
| E290Q | 37% | 63% |
| E290Y | 39% | 61% |

Other mutants that are not included in the table because they are only present in one oligomeric state. S1A, R4K, S10A/C/T, E14D, and Q299A/N produced dimers that were similar to the elution volume of the WT enzyme. On the other hand, R4A/Q, S139A, E290A/D and Q299E produced dimers that have lower elution volume.

**Table S2**: The kinetic parameters of 3CLpro determined at 30 °C and pH 7.0.

| 3CLpro | *k*_cat_ (min^-1^)  **Fold change** | *K*_m_ (µM)  **Fold change** | *k*_cat_/*K*_m_ (µM^-1^ min^-1^)  **Fold change** |
| --- | --- | --- | --- |
| WT | 69 ± 7 | 62 ± 6 | 1.1 ± 0.1 |
| S1A | 101 ± 10  **+1.5** | 94 ± 8  **+1.5** | 1.1 ± 0.1  **+1.0** |
| R4A | 16 ± 6  **–4.3** | 115 ± 3  **+1.9** | 0.14 ± 0.01  **–7.9** |
| S10T | 60 ± 3  **–1.2** | 106 ± 7  **+1.7** | 0.57 ±0.02  **–1.9** |
| K12A | 33 ± 2  **–2.1** | 83 ± 3  **+1.3** | 0.40 ± 0.02  **–2.8** |
| S139A | 36 ± 1  **–1.9** | 95 ± 8  **+1.5** | 0.38 ± 0.03  **–2.9** |
| E166A | 43 ± 6  **–1.6** | 97 ± 7  **+1.6** | 0.44 ± 0.04  **–2.5** |
